# Acute kidney injury in children

**DOI:** 10.1007/s00467-008-1074-9

**Published:** 2009-02-01

**Authors:** Sharon Phillips Andreoli

**Affiliations:** 1 Department of Pediatrics, James Whitcomb Riley Hospital for Children, Indiana University Medical Center, Indianapolis, IN USA; 2 Riley Research Room 230, 699 West Street, Indianapolis, IN 46077 USA

**Keywords:** Acute renal failure, Acute kidney injury, Hypoxic/ischemic injury, Acute tubular necrosis

## Abstract

Acute kidney injury (AKI) (previously called acute renal failure) is characterized by a reversible increase in the blood concentration of creatinine and nitrogenous waste products and by the inability of the kidney to regulate fluid and electrolyte homeostasis appropriately. The incidence of AKI in children appears to be increasing, and the etiology of AKI over the past decades has shifted from primary renal disease to multifactorial causes, particularly in hospitalized children. Genetic factors may predispose some children to AKI. Renal injury can be divided into pre-renal failure, intrinsic renal disease including vascular insults, and obstructive uropathies. The pathophysiology of hypoxia/ischemia-induced AKI is not well understood, but significant progress in elucidating the cellular, biochemical and molecular events has been made over the past several years. The history, physical examination, and laboratory studies, including urinalysis and radiographic studies, can establish the likely cause(s) of AKI. Many interventions such as ‘renal-dose dopamine’ and diuretic therapy have been shown not to alter the course of AKI. The prognosis of AKI is highly dependent on the underlying etiology of the AKI. Children who have suffered AKI from any cause are at risk for late development of kidney disease several years after the initial insult. Therapeutic interventions in AKI have been largely disappointing, likely due to the complex nature of the pathophysiology of AKI, the fact that the serum creatinine concentration is an insensitive measure of kidney function, and because of co-morbid factors in treated patients. Improved understanding of the pathophysiology of AKI, early biomarkers of AKI, and better classification of AKI are needed for the development of successful therapeutic strategies for the treatment of AKI.

## Introduction

Acute kidney injury (AKI) (previously called acute renal failure) is characterized by a reversible increase in the blood concentration of creatinine and nitrogenous waste products and by the inability of the kidney to regulate fluid and electrolyte homeostasis appropriately. There are many causes of AKI, and the more common ones are listed in Table 1. Some causes of AKI, such as rapidly progressive glomerulonephritis (RPGN), may present as AKI but rapidly evolve into chronic kidney disease (CKD). Several renal diseases, such as the hemolytic–uremic syndrome (HUS), Henoch–Schönlein purpura, and obstructive uropathy with associated renal dysplasia, may present as AKI with improvement of renal function to normal or near-normal levels, but the child’s renal function may slowly deteriorate, leading to CKD several months to years later.

**Table 1:** Etiology of common causes of acute kidney injury

Type	Etiology
Pre-renal injury	Decreased true intravascular volume
	Decreased effective intravascular volume
Intrinsic renal disease	Acute tubular necrosis (vasomotor nephropathy)
	Hypoxic/ischemic insults
	Drug induced
	Toxin mediated
	Endogenous toxins—hemoglobin, myoglobin
	Exogenous toxins—ethylene glycol, methanol
	Uric acid nephropathy and tumor lysis syndrome
	Interstitial nephritis
	Drug induced
	Idiopathic
	Glomerulonephritis—RPGN
	Vascular lesions
	Renal artery thrombosis
	Renal vein thrombosis
	Cortical necrosis
	Hemolytic uremic syndrome
	Hypoplasia/dysplasia with or without obstructive uropathy
	Idiopathic
	Exposure to nephrotoxic drugs in utero
Obstructive uropathy	Obstruction in a solitary kidney
	Bilateral ureteral obstruction
	Urethral obstruction

Children with AKI due to hypoxic/ischemic insults, HUS, acute glomerulonephritis and other causes are more likely to demonstrate oliguria or anuria (urine output less than 500 ml/24 h in older children or urine output less than 1 ml/kg per hour in younger children and infants). Children with acute interstitial nephritis, nephrotoxic renal insults including aminoglycoside nephrotoxicity, and contrast nephropathy are more likely to have AKI with normal urine output. The morbidity and mortality rates of non-oliguric AKI are less than those of oliguric renal failure [1–5]. This review will discuss the epidemiology of AKI, the common causes of AKI, the pathophysiology of hypoxia/ischemia-induced AKI, the new aspect of management of AKI, and potential future therapies for AKI. This review will not address fluid and electrolyte management, nutritional therapy, or renal replacement therapy for AKI, as these aspects of AKI will be the topics of forthcoming reviews.

## Epidemiology of acute kidney injury

While the precise incidence and causes of AKI in pediatric patients is unknown, recent studies suggest that the incidence of AKI in hospitalized children is increasing [1–10]. An important cause of AKI in hospitalized children is in the setting of post-cardiac surgery and in children undergoing stem cell transplantation. AKI in such children is frequently multifactorial, with ischemic/hypoxic injury and nephrotoxic insults being important contributors; the pathophysiology of hypoxic ischemic injury and nephrotoxic insults are descried below. No epidemiology studies using an established definition of AKI have been conducted in pediatric patients. As described below, in pre-renal AKI the kidney is intrinsically normal, and renal function promptly returns to normal with restoration of adequate renal perfusion, while, in acute tubular necrosis, the kidney has sustained intrinsic injury which requires repair and recovery before renal function returns to normal. In a large study of adult patients, the incidence of AKI was 209 per million population, and the most common cause of AKI was pre-renal in 21% of patients and acute tubular necrosis in 45% of patients [11]. Similar epidemiologic studies have not been performed in pediatric patients, but hypoxia/ischemia- and nephrotoxin-induced AKI have been shown to be important causes of AKI in neonates, children and adolescents [1–10]. In a study of pediatric patients in a tertiary care center, 227 children received dialysis during an 8-year interval for an overall incidence of 0.8 per 100,000 total population [2]. In a study of neonates, the incidence of AKI ranged from 8% to 24% of newborns, and AKI was particularly common in neonates who had undergone cardiac surgery [3, 10]. Neonates with severe asphyxia had a higher incidence of AKI, while neonates with moderate asphyxia developed AKI less often [3, 7, 8]. Other studies have demonstrated that very low birth weight (less than 1,500 g), a low Apgar score, a patent ductus arteriosus and maternal receipt of antibiotics and nonsteroidal anti-inflammatory drugs was associated with the development of AKI [6]. A low Apgar score and maternal ingestion of nonsteroidal anti-inflammatory drugs has been associated with decreased renal function in preterm infants [6, 12]. The incidence of AKI in newborns in a developing country was 3.9/1,000 live births and 34.5/1,000 newborns admitted to the neonatal unit [7].

Several studies have demonstrated that, in addition to environmental factors, there may be genetic risk factors for AKI in some newborns and children. Several candidate polymorphisms have not been shown to be associated with AKI, while other polymorphisms have been found to be associated with AKI. Polymorphism of the angiotensin-converting enzyme (ACE) gene or the angiotensin receptor gene, with resultant alterations in activity of the renin–angiotensin system, does not appear to play a role in the development of AKI [13]. In studies of newborns, polymorphisms of tumor necrosis factor alpha, interleukin 1b, interleukin 6 and interleukin 10 genes were investigated to determine if polymorphisms of these genes would lead to a more intense inflammatory response and predispose newborns to AKI [14]. The allelic frequency of the individual genes did not differ between newborns with AKI and those without AKI, but the TNFa/IL-6 AG/GC haplotype was present in 26% of newborns who developed AKI compared to 6% of newborns who did not develop AKI. The investigators suggested that the combination of these polymorphisms might lead to a greater inflammatory response and the development of AKI in neonates with infection [13]. As described below, future therapies for AKI might involve strategies to interrupt the inflammatory response. In other studies, the incidence of ACE I/D allele genotypes or the variants of the angiotensin I receptor gene did not differ in neonates with AKI compared to neonates without AKI, but they may be associated with patent ductus arteriosus and heart failure and indirectly contribute to CKD [14, 15]. AKI occurred more commonly in very low birth weight neonates carrying the heat shock protein 72 (1267) GG genetic variation, which is associated with low inducibility of heat shock protein 72 [16]. Given the important role of heat shock proteins in ischemic renal injury, these findings suggest that some neonates are more susceptible to ischemic injury [17]. Future studies of the genetic background of the child at risk for AKI due to medication exposure, toxin exposure, ischemic hypoxic insults or other insults will likely impact on the treatment of the child at risk for AKI and the management of AKI.

## Diagnosis and etiology of acute kidney injury

Table 1 provides a partial listing of the many different and diverse causes of AKI in children. Currently, there is not a uniform definition of AKI in adult and pediatric patients, and AKI is defined in multiple ways, but the majority of definitions of AKI currently in use involve a change in the serum creatinine level. It is accepted that the concentration of serum creatinine is an insensitive and delayed measure of decreased kidney function following AKI. Other biomarkers under investigation include changes in plasma neutrophil gelatinase-associated lipocalin (NGAL) and cystatin C levels and urinary changes in NGAL, interleukin-18 (IL-18) and kidney injury molecule-1 (KIM-1) [18]. As described below, the development, testing and successful implementation of therapeutic strategies in AKI will require the development of sensitive biomarkers, so that therapy can be initiated in a timely manner. As described above, the definition of AKI in adults and pediatric patients has been quite variable. A new classification system entitled the RIFLE criteria (*R* risk for renal dysfunction, *I* injury to the kidney, *F* failure of kidney function, *L* loss of kidney function, and *E* end-stage renal disease) has been proposed as a standardized classification of acute kidney injury in adults [19] and has been adapted for pediatric patients [20]. The pediatric RIFLE (pRIFLE) was found to classify pediatric AKI better and to reflect the course of AKI in children admitted to the intensive care unit (ICU) [20]. The pediatric RIFLE criteria appear to be quite promising for better characterization of AKI and has been validated in children; additional studies are needed to validate this classification further [20]. Further validation and utilization of pRIFLE criteria would allow inter-center comparisons to be made of AKI in children. The RIFLE criteria are utilized by the Acute Kidney Injury Network (AKIN), which is a group of adult nephrologists, pediatric nephrologists, critical care physicians and societal organizations interested in AKI research; the focus of AKIN is to facilitate international, interdisciplinary and intersociety collaborations to ensure progress in the field of AKI [21].

AKI can be divided into pre-renal injury, intrinsic renal disease, including vascular insults, and obstructive uropathies (see Table 1). Some causes of AKI, such as cortical necrosis and renal vein thrombosis, occur more commonly in neonates, whereas HUS is more common in young children, and RPGN generally occurs in older children and adolescents. An important cause of AKI in neonates is exposure to maternal drugs in utero that interfere with nephrogenesis such as angiotensin-converting enzyme inhibitors, angiotensin receptor blockers and nonsteroidal anti-inflammatory drugs [22–25]. The history, physical examination, and laboratory tests such as urinalysis and radiographic studies can establish the likely cause(s) of AKI. In many instances, such as AKI occurring in hospitalized children, multiple factors are likely to be implicated in the etiology of AKI.

## Pre-renal injury

Pre-renal injury occurs when blood flow to the kidney is reduced due to true intravascular volume contraction or to decreased effective blood volume. Since the kidneys are intrinsically normal, pre-renal injury is reversible once the blood volume and hemodynamic conditions have been restored to normal. Prolonged pre-renal injury can result in intrinsic AKI due to hypoxic/ischemic acute tubular necrosis (ATN). The evolution of pre-renal injury to intrinsic renal injury is not sudden, and several compensatory mechanisms maintain renal perfusion when renal hemodynamics are not optimal. When renal perfusion is compromised, the afferent arterioles relax their vascular tone to decrease renal vascular resistance and maintain renal blood flow. During renal hypoperfusion, the intrarenal generation of vasodilatory prostaglandins, including prostacyclin, mediates vasodilatation of the renal microvasculature to maintain renal perfusion. Administration of cyclo-oxygenase inhibitors such as aspirin or nonsteroidal anti-inflammatory drugs can inhibit this compensatory mechanism and precipitate acute renal insufficiency [26]. Similarly, when renal perfusion pressure is low, as in renal artery stenosis, the intraglomerular pressure necessary to drive filtration is, in part, mediated by increased intrarenal generation of angiotensin II to increase efferent arteriolar resistance. Administration of angiotensin-converting enzyme inhibitors in these conditions can eliminate the pressure gradient needed to drive filtration and precipitate AKI [27, 28]. Thus, administration of medications that can interfere with compensatory mechanisms to maintain renal perfusion can precipitate AKI in certain clinical circumstances.

Pre-renal injury results from renal hypoperfusion due to true volume contraction from hemorrhage, dehydration due to gastrointestinal losses, salt-wasting renal or adrenal diseases, central or nephrogenic diabetes insipidus, increased insensible losses, as occurs in burns, and in disease states associated with third space losses, such as sepsis, nephrotic syndrome, traumatized tissue, and capillary leak syndrome. Decreased effective blood volume occurs when the true blood volume is normal or increased but renal perfusion is decreased due to diseases such as congestive heart failure, cardiac tamponade, and hepatorenal syndrome. Whether pre-renal injury is caused by true volume depletion or decreased effective blood volume, correction of the underlying disturbance will return renal function to normal.

Several measures of urinary parameters, including urine osmolality, urine sodium concentration, the fractional excretion of sodium, and the renal failure index, have all been proposed to be used to help differentiate pre-renal injury from hypoxic/ischemic AKI. Hypoxic/ischemic AKI is also called vasomotor nephropathy and/or acute tubular necrosis, since there are early intense vascular constrictions followed by later tubular injury. Renal tubules are working appropriately in pre-renal injury and are able to conserve salt and water appropriately, whereas, in vasomotor nephropathy, the tubules have progressed to irreversible injury and are unable to conserve salt appropriately [1–5, 29, 30]. During pre-renal injury the tubules respond to decreased renal perfusion by appropriately conserving sodium and water such that the urine osmolality is greater than 400–500 mosmol/l, urine sodium is less than 10–20 mEq/l, and the fractional excretion of sodium is less than 1%.

Because the renal tubules in newborns and premature infants are relatively immature compared with those in older infants and children, the corresponding values suggestive of renal hypoperfusion are urine osmolality greater than 350 mosmol/l, urine sodium less than 20–30 mEq/l, and fractional excretion of sodium of less than 2.5% [29, 30]. When the renal tubules have sustained injury, they cannot conserve sodium and water appropriately, so that the urine osmolality is less than 350 mosmol/l, the urine sodium is greater than 30–40 mEq/l, and the fractional excretion of sodium is greater than 2.0%. The use of these values to differentiate pre-renal injury from ATN requires that the patient have normal tubular function initially. However, newborns with immature tubules and children with pre-existing renal disease or salt-wasting renal adrenal disease, as well as other diseases, might have pre-renal injury with urinary indices suggestive of ATN but might, in reality, have pre-renal injury. Thus, it is essential that we consider the state of the function of the tubules before the potential onset that might precipitate vasomotor nephropathy/ATN, so that ascribing pre-renal injury to vasomotor nephropathy/ATN does not occur. In addition, the fractional excretion of sodium is often difficult to interpret in patients who have received diuretic therapy.

### Intrinsic renal disease

#### Hypoxic/ischemic acute kidney injury

Hypoxic/ischemic AKI is characterized by early vasoconstriction followed by patchy tubular necrosis. Recent studies suggest that the vasculature of the kidney may play a role in acute injury and chronic injury as well, and the endothelial cell has been identified as a target of injury. Peritubular capillary blood flow has been shown to be abnormal during reperfusion, and there is also loss of normal endothelial cell function in association with distorted peritubular pericapillary morphology and function [31, 32]. The mechanism of cellular injury in hypoxic/ischemic AKI is not known, but alterations in endothelin or nitric oxide regulation of vascular tone, ATP depletion and alterations in the cytoskeleton, changes in heat shock proteins, initiation of the inflammatory response and the generation of reactive oxygen and nitrogen molecules may each play a role in cell injury [31–48].

Nitric oxide is a vasodilator produced from endothelial nitric oxide synthase (eNOS), and nitric oxide helps regulate vascular tone and blood flow in the kidney [37, 38]. Recent studies suggest that loss of normal eNOS function occurs following ischemic/hypoxic injury which could precipitate vasoconstriction [37]. In contrast, inducible nitric oxide synthase (iNOS) activity increases following hypoxic/ischemic injury, and iNOS can participate in the generation of reactive oxygen and nitrogen molecules. Inducible nitric oxide synthase, with the generation of toxic nitric oxide metabolites including peroxynitrate, has been shown to mediate tubular injury in animal models of acute kidney injury [38, 39]. Endothelin (ET) peptides are potent vasoconstrictors that have also been shown to play a role in the pathogenesis of AKI in animal models [40–42]. In rat models of AKI, circulating levels of endothelin-1 and tissue expression of endothelin-1 protein levels was substantially increased, while ET(A) and ET(B) receptor gene expression was also increased after ischemic injury [41]. Endothelin receptor agonist for the A receptor have been shown to be shown to decrease AKI in animal models [42]. Thus, alterations in the balance of vasoconstrictive and vasostimulatory stimuli are likely to be involved in the pathogenesis of hypoxic/ischemic AKI.

An initial response to hypoxic/ischemic AKI is ATP depletion, which leads to a number of detrimental biochemical and physiologic responses, including disruption of the normal cytoskeletal organization with loss of the apical brush border and loss of polarity with Na+K+ATPase localized to the apical as well as the basolateral membrane [43]. This has been shown in several animal models of AKI, and it has also been shown in human renal allografts that loss of polarity with mislocation of Na+K+ATPase to apical membrane contributes to kidney dysfunction in transplanted kidneys [44]. Reactive oxygen molecules are also generated during reperfusion and can contribute to tissue injury [34]. While tubular cells and endothelial cells are susceptible to injury by reactive oxygen molecules, studies have shown that endothelial cells are more sensitive to oxidant injury than tubular epithelial cells are [35]. Other studies have shown an important role for heat shock protein in modifying the renal response to ischemic injury as well as playing a role in promoting recovery of the cytoskeleton following AKI [45].

In children with multiorgan failure, the systemic inflammatory response is thought to contribute to AKI as well as other organ dysfunction by the activation of the inflammatory response, including increased production of cytokines and reactive oxygen molecules, activation of polymorphonuclear leukocytes (PMNs), and increased expression of leukocyte adhesion molecules [46]. Reactive oxygen molecules can be generated by several mechanisms including activated PMNs, which may cause injury by the generation of reactive oxygen molecules including superoxide anion, hydrogen peroxide, hydroxyl radical, hypochlorous acid, and peroxynitrite, or by the release of proteolytic enzymes. Myeloperoxidase from activated PMNs converts hydrogen peroxide to hypochlorous acid, which may react with amine groups to form chloramines; each of these can oxidize proteins, DNA, and lipids, resulting in substantial tissue injury [34, 47]. Leukocyte endothelial cell adhesion molecules have been shown to be unregulated in ATN, and administration of anti-adhesion molecules can substantially decrease renal injury in animal models of ATN [36]. As described below, several animal models have shown that interference with the inflammatory response may be the future therapy for hypoxic/ischemic AKI. Studies of humans with AKI have demonstrated an increased evidence of oxidation of proteins reflecting oxidant stress [48].

In the past it was thought that recovery from hypoxic/ischemic and nephrotoxic AKI was complete with return of renal function to normal, but recent studies have shown that recovery may be partial and that the patient is at higher risk for later chronic kidney disease [31]. In addition, hypoxic/ischemic insults can result in physiologic and morphological alterations in the kidney that can lead to kidney disease at a later time [49].

#### Nephrotoxic acute kidney injury

Medications associated with AKI, at least in part due to toxic tubular injury, include aminoglycoside antibiotics, intravascular contrast media, amphotericin B, chemotherapeutic agents such as ifosfamide and cisplatin, acyclovir, and acetaminophen, while other medications have been implicated less commonly. Aminoglycoside nephrotoxicity typically presents with nonoliguric AKI, with urinalysis showing minimal urinary abnormalities. The incidence of aminoglycoside antibiotic nephrotoxicity is related to the dose and duration of the antibiotic therapy as well as the level of renal function prior to the initiation of aminoglycoside therapy. The etiology is thought to be related to the lysosomal dysfunction of proximal tubules and is reversible once the aminoglycoside antibiotics have been discontinued. However, after the aminoglycoside has been discontinued, the serum creatinine may continue to increase for several days due to ongoing tubular injury from continued high parenchymal levels of the aminoglycoside. Cisplatin, ifosfamide, acyclovir, amphotericin B, and acetaminophen are also nephrotoxic and may precipitate AKI. Several other drugs have also been associated with AKI, but the incidence of AKI with other drugs is less common. Hemolysis and rhabdomyolysis from any cause can result in sufficient hemoglobinuria or myoglobinuria to induce tubular injury and precipitate AKI. The mechanisms of injury are complex but may be related to vasoconstriction, precipitation of the pigments in the tubular lumen, and/or heme-protein–induced oxidant stress [50].

### Uric acid nephropathy and tumor lysis syndrome

Children with acute lymphocytic leukemia and B-cell lymphoma are at the highest risk of AKI due to uric acid nephropathy and/or the tumor lysis syndrome [51, 52]. Although the pathogenesis of uric acid nephropathy is complex, a potentially important mechanism of injury is related to the precipitation of crystals in the tubules, which obstruct urine flow, or in the renal microvasculature, which obstruct renal blood flow [51, 52]. A common cause of AKI in leukemia is the development of the tumor lysis syndrome during chemotherapy [51–53]. Therapy with allopurinol will limit the increased excretion of uric acid with chemotherapy, but allopurinol therapy will result in markedly increased excretion of uric acid precursors, including hypoxanthine and xanthine, and precipitate xanthine nephropathy [51, 54]. Xanthine is less soluble than uric acid, and precipitation of hypoxanthine and xanthine may play a role in the development of AKI during the tumor lysis syndrome [54]. Rasburicase is a recombinant form of urate oxidase that catalyzes uric acid to allantoin, which is five times more soluble than uric acid [55]. Rasburicase has been shown to be effective and well tolerated in the prevention of renal failure in pediatric patients with tumor lysis syndrome [55]. AKI during tumor lysis syndrome can also result from extreme hyperphosphatemia from rapid breakdown of tumor cells and the precipitation of calcium phosphate crystals [56].

### Acute interstitial nephritis

Acute interstitial nephritis (AIN) may cause renal failure as a result of a reaction to a drug or due to idiopathic AIN. Children with AIN may have rash, fever, arthralgias, eosinophilia, and pyuria with or without eosinophiluria. Medications commonly associated with AIN include methicillin and other penicillin analogs, cimetidine, sulfonamides, rifampin, nonsteroidal anti-inflammatory drugs, and proton pump inhibitors, whereas other drugs have been associated with AIN less commonly [57]. AIN associated with nonsteroidal anti-inflammatory drugs may also present with high-grade proteinuria and nephrotic syndrome. Specific therapy for AIN includes withdrawal of the drug implicated in causing the AIN. In addition, corticosteroids may aid in the resolution of the renal failure [57].

### Rapidly progressive glomerulonephritis

Any form of glomerulonephritis in its most severe degree can present with AKI and RPGN. The clinical features include hypertension, edema, hematuria that is frequently gross, and a rapidly rising levels of blood urea nitrogen (BUN) and creatinine. The characteristic pathological finding in RPGN is extensive crescent formation. RPGN due to postinfectious glomerulonephritis typically does not lead to CKD, while other glomerulonephritides, such as antineutrophil cytoplasmic antibody (ANCA)-positive glomerulonephritis, Goodpasture’s syndrome, and idiopathic RPGN, typically present with AKI and may quickly evolve into CKD, with or without therapy. Serological tests including an antinuclear antibody (ANA), ANCA, anti–glomerular basement membrane (GBM) titers, and complement studies are required to evaluate the etiology of the RPGN. Because specific therapy will depend on the pathological findings, a biopsy should be performed relatively quickly when a child presents with clinical characteristics suggestive of RPGN.

### Vascular insults

Cortical necrosis as a cause of AKI is much more common in young children, particularly in neonates. Cortical necrosis is associated with hypoxic/ischemic insults due to perinatal anoxia, placenta abruption, and twin–twin or twin–mother transfusions, with resultant activation of the coagulation cascade. Children and newborns with cortical necrosis usually have gross or microscopic hematuria and oliguria and may have hypertension as well. In addition to laboratory features of elevated levels of BUN and creatinine, thrombocytopenia may also be present, due to the microvascular injury. Radiographic features include normal findings of renal ultrasound in the early phase, and ultrasound in the later phases may show that the kidney has undergone atrophy and has substantially decreased in size. The prognosis for cortical necrosis is much worse than that for ATN. Children with cortical necrosis may partially recovery or not recover at all. HUS is a common cause of AKI in children and leads to substantial morbidity and mortality rates and long-term complications that may not become apparent until adulthood [58]. While an important cause of AKI in children, HUS will not be further discussed here, as a review of HUS has recently been published [58].

### Obstructive uropathy

Obstruction of the urinary tract can cause acute kidney injury if the obstruction occurs in a solitary kidney, if it involves the ureters bilaterally, or if there is urethral obstruction. Obstruction can result from congenital malformations such as posterior urethral valves, bilateral ureteropelvic junction obstruction or bilateral obstructive ureteroceles. Acquired urinary tract obstruction can result from passage of kidney stones or, rarely, from tumors. It is important to evaluate for obstruction, since the management is to relieve the obstruction promptly.

## Management of acute kidney injury in children

### Preventive measures

Two studies from different geographic regions of Nigeria demonstrated that the most common cause of AKI in children was volume depletion and that the AKI was due to preventable cause [59, 60]. Since dialytic resources were scare, the mortality rate in these studies was quite high [59, 60]. Thus, on a global scale, the prevention of AKI is likely to have a larger impact on mortality rates than other measures.

Intravenous infusion of theophylline, given to severely asphyxiated neonates within the first hour of birth, was associated with improved fluid balance, creatinine clearance, and reduced serum creatinine levels and had no effects on neurological and respiratory complications [61–63]. Adenosine is a potent vasoconstrictor that is released from the catabolism of ATP during ischemia; the potential mechanisms that theophylline could protect from AKI could be the blocking of the adenosine receptor. Other studies of asphyxiated neonates also demonstrated improved renal function and decreased excretion of beta-2 microglobulin in neonates given theophylline within one hour of birth [61–63]. However, the clinical significance of the improved renal function was not clear, and the incidence of persistent pulmonary hypertension was higher in the neonates who had received theophylline group. Additional studies are needed to determine the significance of these findings and the potential side effects of theophylline.

### Diuretics and dopamine receptor agonist

Diuretics and ‘renal-dose’ dopamine are commonly used to prevent or limit AKI. There have been several clinical studies using mannitol, diuretics, and ‘renal-dose’ dopamine for AKI [64–71]. The stimulation of urine output eases management of AKI, but conversion of oliguric to nonoliguric AKI has not been shown to alter the course of renal failure [64]. Furosemide may increase the urine flow rate to decrease intratubular obstruction and will inhibit Na-K-ATPase, which will limit oxygen consumption in already damaged tubules with a low oxygen supply. In a randomized controlled trial, two groups of adult patients with AKI requiring dialysis were given furosemide therapy or placebo; diuresis was achieved in a significantly shorter time in the group that received furosemide than in the group that had received placebo [64]. However, there was not a difference in the number of dialysis sessions, time on dialysis, or patients’ survival [64]. In patients who do respond to diuretic therapy with an increase in urine output, continuous infusions may be associated with less toxicity than bolus administration [65]. A retrospective study actually demonstrated that the use of diuretics in AKI was associated with adverse outcomes [66]. Since high doses of furosemide can cause ototoxicity, continued use in individual patients with AKI needs to take into consideration the risks and potential benefits or lack of benefits.

The use of ‘renal-dose’ dopamine (0.5 μg/kg per minute to 3–5 μg/kg per minute) to improve renal perfusion following an ischemic insult has become very common in intensive care units. While dopamine increases renal blood flow by promoting vasodilatation and may improve urine output by promoting natriuresis, there have been no definitive studies to demonstrate that low doses of dopamine are effective in decreasing the need for dialysis or improve survival times in patients with AKI [67–71]. In fact, a placebo controlled randomized study of low doses of dopamine in adult patients demonstrated that low doses were not beneficial and did not confer clinically significant protection from renal dysfunction [67]. Other studies have demonstrated that ‘renal-dose’ dopamine is not effective in the therapy of AKI, and one study demonstrated that low doses worsened renal perfusion and renal function [69]. Three separate meta-analyses have shown no benefit of dopamine in AKI [68, 70, 71]. Fenoldopam is a potent, short-acting, selective, dopamine-1 receptor agonist that decreases vascular resistance while increasing renal blood flow [72]. A recent meta-analysis of 16 trials of fenoldopam concluded that therapy with fenoldopam decreased the incidence of acute kidney injury, decreased the need for renal replacement therapy, decreased ICU stay and decreased the number of deaths from any cause [73]. Fenoldopam has been used in a few children with acute kidney injury, including two children receiving therapy with a ventricular-assist device as a bridge to cardiac transplantation; therapy with fenoldopam was thought to avoid the need for renal replacement therapy in one child [74]. Additional studies utilizing fenoldopam need to be performed on children with acute kidney injury.

### Therapies to decrease injury and promote recovery

While there is no current specific therapy to prevent renal injury or promote recovery in human ATN, several potential therapies are being studied, and future management of AKI may also include antioxidant, anti-adhesion molecule therapy and the administration of vascular mediators or mesenchymal stem cells to prevent injury and/or promote recovery [35, 36, 75–77]. Several different therapies have been shown to prevent, decrease or promote recovery in animal models of AKI. Melanocyte-stimulating hormone (MSH) has anti-inflammatory activity and has been shown to protect renal tubules from injury [75]. In other animal models of AKI, post-ischemic infusion of growth factors, including insulin-like growth factor-1 (IGF-1), epidermal growth factor, and hepatocyte growth factor, resulted in accelerated recovery of renal function, less severe histological alterations and decreased mortality rates [75–77]. Scavengers of free radicals and reactive oxygen and nitrogen molecules, as well as anti-adhesion molecules, have been shown to decrease the degree of injury in animal models of AKI [77]. Recently, very interesting studies have also demonstrated that multipotent mesenchymal stem cells (MSCs) may have a role in promoting recovery from AKI in animal models [78].

Despite the promise of animal models of intervention in AKI, clinical studies in humans have been largely disappointing, including studies that utilized anaritide (atrial natriuretic peptide) and IGF-1 [79, 80]. Since therapy in these studies was initiated when renal failure was well established, it is likely that the opportunity to intervene and have an impact on the recovery from AKI had been missed [81, 82]. As mentioned previously, the development and testing of interventions for AKI will require the development of early biomarkers of injury that are much more sensitive than serum concentrations of creatinine.

## Prognosis of acute kidney injury

The prognosis of AKI is highly dependent on the underlying etiology of the AKI. Children who have AKI as a component of multisystem failure have a much higher mortality rate than children with intrinsic renal disease such as HUS, RPGN, and AIN. Recovery from intrinsic renal disease is also highly dependent on the underlying etiology of the AKI. Children with nephrotoxic AKI and hypoxic/ischemic AKI usually recover normal renal function. In the past it has been thought that such patients are at a low risk for late complications, but several recent studies have demonstrated that chronic kidney disease can evolve from AKI [83–88]. Children who have suffered substantial loss of nephrons, as in HUS or RPGN, are at risk for late development of renal failure long after the initial insult has occurred. Several studies in animal models have documented that hyperfiltration of the remnant nephron may eventually lead to progressive glomerulosclerosis of the remaining nephrons. Thus, children who have had cortical necrosis during the neonatal period and whose renal function has recovered, or children with an episode of severe Henoch–Schönlein purpura or HUS, are clearly at risk for the late development of renal complications. Such children need life-long monitoring of their renal function and blood pressure, and life-long urinalyses.

As described above, it has been thought that acute kidney injury due to hypoxic/ischemic and nephrotoxic insults were reversible, with a return of renal function to normal. However, recent studies have demonstrated that hypoxic/ischemic and nephrotoxic insults can lead to physiologic and morphologic alterations in the kidney that may lead to kidney disease at a later time [50]. Studies of adults demonstrate that CKD may evolve from AKI [83, 84]. Thus, acute kidney injury from any cause can be a concern for later kidney disease. Importantly, acute kidney injury is likely to be especially deleterious when the kidney has not yet grown to adult size and/or before the full complement of nephrons have developed. Since nephrogenesis is not complete until approximately 34 weeks’ gestation, acute kidney injury during this interval might lead to a decreased number of nephrons, and, indeed, studies have suggested that acute kidney injury during nephrogenesis results in decreased numbers of nephrons and subsequent glomerulomegaly [86]. Acute kidney injury in the full-term neonate is also associated with later kidney disease [88]. In one study of six older children with a history of AKI not requiring dialysis in the neonatal period, only two were healthy, three had chronic renal failure and one was on dialysis. Studies on older children have also shown that AKI leads to CKD in a higher percentage of children than was previously appreciated [85]. In a prospective study of renal insufficiency in children undergoing bone marrow transplantation, the incidence of acute renal insufficiency was high and was predictive of chronic renal insufficiency. Of those who survived, 11% developed chronic kidney disease, and AKI was the sole predictor of chronic kidney disease [89]. Thus, children with a history of AKI from any cause need long-term follow-up.

## Questions (answers appear following the reference list)

1. Epidemiology studies of AKI in children has shown that
  - the incidence of AKI is decreasing: true or false
  - genetic factors may play a role in the susceptibility to AKI: true or false
  - hypoxic/ischemic AKI is a rare cause of AKI in children: true or false
  - nonsteroidal anti-inflammatory therapy is a risk factor for AKI in newborns: true or false
2. Diagnosis and etiology of AKI in children
  - the definition of AKI in children is well established: true or false
  - the serum creatinine is a sensitive marker of AKI: true or false
  - in utero exposure to ACE inhibitors is associated with AKI in neonates: true or false
  - AKI in hospitalized children may be the result of multiple etiologies: true or false
  - urinary sodium concentration is a reliable indicator of pre-renal versus hypoxic/ischemic AKI in premature newborns: true or false
3. Hypoxic/ischemic AKI
  - alterations in blood flow are not thought to play a role in hypoxic/ischemic AKI: true or false
  - reactive oxygen molecules generated by activated PMNs may play a role in hypoxic/ischemic ATN: true or false
  - ATP depletion is an early response to hypoxic/ischemic AKI: true or false
  - hypoxic/ischemic AKI is always reversible and has no long-term consequences: true or false
4. Management of AKI in children
  - diuretic therapy in AKI shortens hospital stay, decreases the need for dialysis therapy and decreases mortality rates: true or false
  - ‘renal-dose’ dopamine therapy has not been shown to be effective in adult patients: true or false
  - prevention of AKI is important on a global scale in lowering the number of deaths from AKI: true or false
5. Prognosis of AKI
  - neonates, infants, children and adolescents who have recovered from AKI do not need long-term follow up: true or false
  - AKI that occurs before nephrogenesis is complete may lead to later CKD: true or false
